# Optimization of purification process and thermal stability of lactoferrin from camel milk

**DOI:** 10.1016/j.fochx.2026.104616

**Published:** 2026-10-16

**Authors:** Yuying Liu, Ying Liu, Hongyi Zhang, Minaer Huanbieke, Lixiang Ma, Chunyan Ran, Yaqi Cheng, Jie Yang, Haitao Yue, Yang Qu

**Affiliations:** **College of Life Science and Technology**, **Xinjiang University, Urumqi** 830046, China

**Keywords:** Xinjiang Bactrian camel milk, Camel lactoferrin, Purification, Thermal stability

## Abstract

To expand the application of camel lactoferrin (CLF) in thermally processed foods, this study optimized cation-exchange chromatography to investigate its thermally induced conformational evolution. Under optimal conditions (flow rate 1 mL/min, pH 7.0, impurity elution salt concentration containing 0.3 M NaCl), CLF with purity of 90.13% ± 1.06% was obtained with good repeatability (RSD < 5%) for preparation and quantitative analysis. Compared with bovine lactoferrin (BLF), CLF exhibits divergent lobe thermostability. Fluorescence and UV–vis spectra confirm severer unfolding and hydrophobic exposure in CLF at 95 °C, whereas DLS reveals weaker aggregation, showing inconsistent trends between unfolding and aggregation. Molecular dynamics simulations demonstrate CLF adopts a compact internally disordered conformation. Strengthened intramolecular salt bridges and hydrophobic interactions shield hydrophobic sites and inhibit aggregation. This study provides process-optimization ideas and structural insights for the application of CLF in thermally processed foods and the high-value utilization of camel milk resources.

## Highlights

• Established an optimized purification process for CLF with key parameters defined.
• Across 15 s–10 min at 72 °C and 95 °C, CLF showed minimal changes at 72 °C but time-amplified structural changes at 95 °C.
• CLF undergoes pronounced unfolding yet exhibits suppressed aggregation at high temperature.
• Provided a theoretical basis for applying CLF in thermally processed food products.

## Graphical abstract

**Figure:**
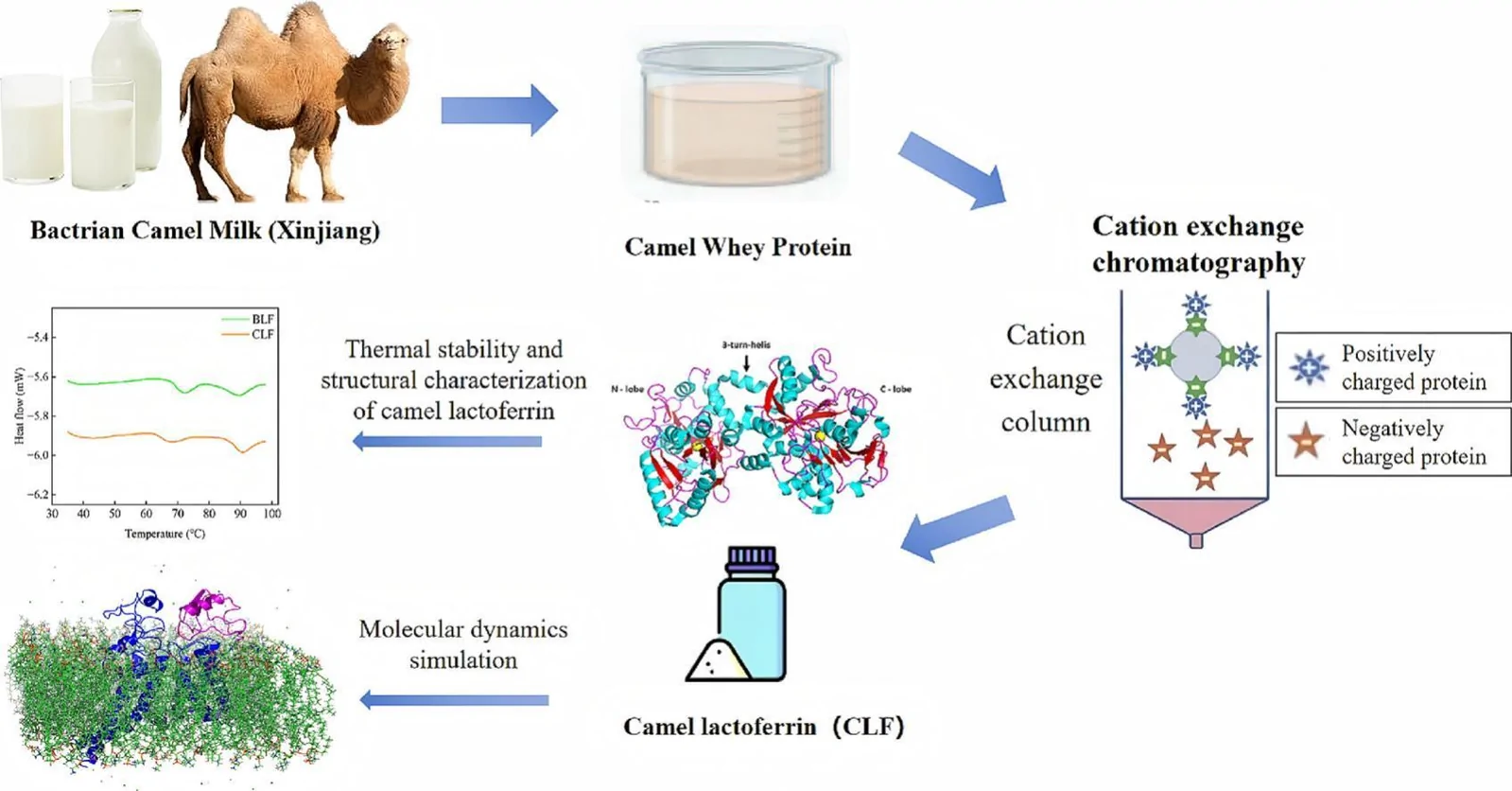

## 1 Introduction

Camels represent vital livestock resources that safeguard local food security in arid and semi-arid regions, supplying sustainable nutritional milk sources for residents inhabiting dryland zones. Camel milk possesses unique nutritional and bioactive properties that distinguish it from bovine milk, among which camel lactoferrin (CLF) is one of the core functional components. As a viable alternative to bovine lactoferrin (BLF), CLF exhibits favorable antiviral (Khan, Khan, et al., 2021) and anti-tumour activities (He et al., 2009), and therefore has promising application prospects in the food and pharmaceutical industries.

CLF differs from BLF with respect to molecular weight, isoelectric point, and concentration in milk. Specifically, CLF has a molecular weight of approximately 75-78 kDa, slightly lower than that of BLF (78–80 kDa), and an isoelectric point ranging from 8.5 to 9.0, while BLF displays an isoelectric point range of 8.0–9.0 (Mahala et al., 2022; Wang et al., 2019). Furthermore, the lactoferrin concentration in camel milk is 2.00–6.00 mg/mL, while that in bovine milk is 0.10–0.40 mg/mL (Du et al., 2017). Although these physicochemical distinctions between CLF and BLF facilitate the optimization of purification protocols and investigations into thermal response behavior, the absence of efficient large-scale purification techniques and limited understanding of thermal stability mechanisms restrict its industrial development and application.

Current mainstream techniques for lactoferrin purification are classified into three types: salting-out, ion exchange chromatography (IEC) and affinity chromatography (Chen et al., 2023; Franco et al., 2018). Salting-out is easy to operate and low-cost for scale-up, yet it suffers from poor separation selectivity and delivers protein products with low purity (Zhang et al., 2024). Heparin affinity chromatography can yield high-purity lactoferrin with intact native conformation via specific binding interactions, whereas the expensive ligands and low processing throughput limit its application only to laboratory-scale preparation of reference standards and mass spectrometry samples (Le Parc et al., 2014; Li et al., 2019). As a subtype of IEC, cation-exchange chromatography (CEX) selectively captures lactoferrin from whey through electrostatic interactions and has become a well-established process for large-scale production of BLF (Tomita et al., 2009). For example, Jia et al. (2021) isolated BLF with a purity of 95% from bovine milk using CEX. Existing purification protocols for CLF have notable drawbacks. Mahala et al. (2022) established a pH-controlled ammonium sulfate fractionation method with low raw material cost and simple operation, but this approach co-precipitates large amounts of serum albumin impurities, and no quantitative purity data was reported in their research. Although heparin affinity chromatography enables high-purity CLF preparation (Li et al., 2019), its high cost and low throughput make it unsuitable for industrial scale-up. BLF and CLF belong to homologous transferrin proteins with highly conserved spatial structures, so the electrostatic capture mechanism of CEX is theoretically applicable to the separation and enrichment of CLF. Nevertheless, efficient CEX protocols tailored to camel milk matrices for preparing high-purity CLF remain lacking, making it difficult to fully explore the inherent bioactivities and functional application potential of CLF.

Thermal treatment acts as an indispensable unit operation in dairy manufacture to eliminate pathogenic microorganisms and extend product shelf life, yet heating can alter the spatial conformation of lactoferrin and compromise its biological activity (Li et al., 2021; Liu et al., 2020). Existing evidence has demonstrated that thermal stimulation triggers conformational unfolding and exposure of internal hydrophobic residues in BLF (Lu et al., 2022a; Wang et al., 2024). As illustrated in Lu et al. (2022b) subjected BLF to representative industrial heating regimens including 63 °C for 30 min and 72 °C for 15 s. Via circular dichroism spectroscopy and surface hydrophobicity determination, they verified that high temperature reduces the α-helical proportion of BLF, induces protein aggregation, and abolishes its osteogenic capacity. J. Lu et al. (2025) further simulated lactose-containing milk powder processing with a series of short-time heating treatments ranging from 75 °C to 130 °C, and found that thermal processing triggers lactosylation modification on lysine residues of BLF, which disrupts its secondary structure and impairs digestibility. Collectively, the conformational evolution and activity loss profiles of BLF under various temperature-time combinations have been comprehensively characterized.

In recent years, spectroscopy combined with chemometrics has been widely used to evaluate camel milk quality and detect adulteration, yet systematic investigations into the thermal stability of CLF remain scarce; no quantitative correlations between industrial heating parameters and CLF conformational shifts have been established, and insufficient basic data limits our understanding of CLF thermal responsiveness and its application in thermally processed dairy products. Against this research gap, we first establish an optimized, CEX purification procedure for camel milk-derived CLF via single-factor experiments and response surface methodology (RSM). A comprehensive panel of characterization techniques, including DSC, CD, intrinsic fluorescence, UV–Vis spectroscopy, DLS and molecular dynamics (MD) simulations, is integrated to compare the thermal behaviors of CLF and BLF. Drawing on prior thermal processing research (J. Lu et al., 2023; Lu et al., 2022b), we adopt two industrial heating regimes (72 °C for conventional pasteurization and 95 °C for intensified pasteurization) paired with three typical holding durations (15 s, 2 min, 10 min) representing short, medium and long industrial heating treatments to systematically elucidate CLF's conformational evolution under different thermal severities. Collectively, this work seeks to construct a transferable CLF purification workflow, decipher CLF thermal denaturation under practical dairy processing conditions, and provide experimental references for large-scale LF manufacture and thermal process optimization, which further facilitates the high-value exploitation of camel milk resources.

## 2 Materials and methods

### 2.1 Materials

The camel milk and bovine milk used in this experiment were collected from the Baotu Camel Breeding Base in Shuimogou District, Urumqi City, Xinjiang. All raw milk samples were stored at −80 °C for subsequent analysis.

The main experimental reagents and instruments were as follows: CM-Sepharose Fast Flow cation-exchange resin (Xi'an Lanxiao Technology Co., Ltd.), AKTA purifier protein purification system (Cytiva Biotechnology Co., Ltd.), 0.22 μm aqueous microporous filter membrane (Merck, Germany). Hydrochloric acid, sodium dihydrogen phosphate, disodium hydrogen phosphate, sodium chloride, sodium hydroxide and absolute ethanol were all supplied by Tianjin Zhiyuan Chemical Reagent Co., Ltd. All chemicals were of analytical grade. Unless otherwise stated, all experiments were conducted in triplicate.

### 2.2 Purification of lactoferrin from camel milk

#### 2.2.1 Extraction and purification of camel lactoferrin

CLF was isolated from Xinjiang Bactrian camel milk following the workflow illustrated in Fig. S1.

Raw milk was centrifuged at 4532 ×*g* and 4 °C for 20 min to remove fat. Skim milk pH was adjusted to 4.6 and incubated at 40 °C for 30 min, followed by a second centrifugation under identical conditions to precipitate caseins. Recovered whey was readjusted to its native pH and filtered using a 0.22 μm aqueous membrane (Yao et al., 2024). Clarified whey was loaded onto a 5 mL pre-packed CM Sepharose Fast Flow cation-exchange chromatography (CIEX) column (binding capacity: 100 mg protein/mL medium). Buffer A (0.02 M PBS, pH 7.0) and Buffer B (0.02 M PBS containing 1 M NaCl, pH 7.0) served as mobile phases. Samples (1 mL injection) were separated at a flow rate of 1.0 mL/min, and protein absorbance was monitored at 280 nm. After column equilibration and sample loading, weakly bound impurities were washed with 40% Buffer B (0.4 M NaCl). A linear gradient from 40% to 100% Buffer B and subsequent isocratic elution with 100% Buffer B were applied to collect crude CLF.

#### 2.2.2 Preparation of camel lactoferrin standards

CLF reference standards and BLF were prepared via heparin affinity chromatography (Gao et al., 2025; Liu, 2022). Purity characterization of the CLF standard and detailed calibration curve construction procedures using an AKTA Purifier are provided in Supplementary Materials (Fig. S5). The purified standard was further identified by ultra-high-performance liquid chromatography (Waters ACQUITY UPLC) and mass spectrometry (Thermo Fisher Scientific Orbitrap Astral).

#### 2.2.3 Purity assessment of camel lactoferrin

The purity of CLF was assessed by sodium dodecyl sulfate-polyacrylamide gel electrophoresis (SDS-PAGE) using a 5% stacking gel and a 12% resolving gel. The electrophoresis was run at 80 V for stacking gel and 120 V for resolving gel, followed by Coomassie Brilliant Blue R-250 staining. Quantification of CLF was performed by ultra-high-performance liquid chromatography (UPLC) equipped with an ACQUITY UPLC Protein BEH C4 column (300 Å, 1.7 μm, 2.1 mm × 100 mm). Mobile phase A was ultrapure water containing 0.1% trifluoroacetic acid, and mobile phase B was acetonitrile containing 0.1% trifluoroacetic acid; the detection wavelength was set at 214 nm.

#### 2.2.4 Optimization of ion exchange chromatography purification process for camel lactoferrin

##### 2.2.4.1 Single-factor experiments

A single-factor experimental design was employed to investigate the effects of buffer flow rate, Buffer pH, and wash salt concentration on the purification efficiency of CLF. The parameter ranges tested were as follows: flow rate of 0.5–1.5 mL/min, Buffer A pH of 6.0–8.0, and impurity washing salt concentration of 0.1–0.5 M NaCl. For each factor, only that parameter was varied while all other conditions were held constant (Table 1).

**Table 1:** Single-factor experimental design.

Variable	Tested levels	Constant conditions
Flow rate (mL/min)	0.5, 0.75, 1.0, 1.25, 1.5	Buffer A: 20 mM phosphate buffer (pH 7.0)
		Stepwise elution: 30% Buffer B (0.3 M NaCl) for impurity washing, followed by 100% Buffer B (1 M NaCl) to elute target CLF
Buffer pH	6.0, 6.5, 7.0, 7.5, 8.0	Flow rate: 1 mL/min
		Buffer A: 20 mM phosphate buffer
		Stepwise elution: 30% Buffer B (0.3 M NaCl) for impurity washing, followed by 100% Buffer B (1 M NaCl) to elute target CLF
Impurity Elution Concentration (%)	10,20,30,40,50(0.1,0.2,0.3,0.4,0.5 M NaCl)	Buffer A: 20 mM phosphate buffer (pH 7.0)
		Flow rate: 1 mL/min

All experiments were carried out on CM-Sepharose Fast Flow cation exchange columns. For each run, 1 mL camel whey protein solution filtered through a 0.22-μm microporous membrane was loaded onto the column.Buffer A refers to 20 mM phosphate equilibrium buffer without NaCl; Buffer B is prepared by adding 1 M NaCl to Buffer A, and the pH of Buffer B was consistent with the corresponding Buffer A in each experimental group.

##### 2.2.4.2 Response surface optimization

Based on single-factor experimental data, Design-Expert software (Version 13.0, Stat-Ease, Minneapolis, USA) was utilized to design the Box-Behnken experiment, with CLF purity (Y) set as the response value to conduct response surface optimization analysis (Table 2).

**Table 2:** Response surface factor levels.

Level	A	B	C
	Flow rates	pH	Impurity Elution Concentration
	(mL/min)	\	(%)
-1	0.75	6.5	20%
0	1.0	7.0	30%
1	1.25	7.5	40%

### 2.3 Thermal stability analysis and structural characterization of lactoferrin

#### 2.3.1 Determination of iron saturation

According to (Wang et al., 2015), the iron saturation of CLF and BLF was determined via absorbance at 465 nm and 280 nm with minor modifications. Filtered 10 mg/mL protein solutions in 0.15 mol/L NaCl were diluted to 1 mg/mL. After 20 min preheating and baseline calibration, A₁ (465 nm, 10 mg/mL) and A₂ (280 nm, 1 mg/mL) were recorded separately for calculation. The iron saturation was calculated using the following formula:

(1) $$\mathrm{Iron saturation}=\frac{A_{1}\times 1.3}{A_{2}\times 0.48}\times 100\%$$

#### 2.3.2 Thermal stability determination by differential scanning calorimetry

BLF and CLF were diluted to 10 mg/mL using 20 mM Buffer A (pH 7.0). A 10 μLaliquot of each sample was transferred into an aluminum crucible, sealed and loaded into the sample chamber, with an empty aluminum crucible as the reference. Measurements were carried out under nitrogen atmosphere from 37 to 100 °C at a heating rate of 10 °C/min (Jennings et al., 2024).

#### 2.3.3 Heat-treated lactoferrin

BLF and CLF were diluted to 0.2 mg/mL with Milli-Q water and aliquoted into tubes. Samples were heated in a water bath at 72 °C and 95 °C for 15 s, 2 min or 10 min, then rapidly cooled to 4 °C in ice water. Unheated native samples subjected to identical handling served as controls.

#### 2.3.4 Fluorescence spectroscopy analysis

Intrinsic fluorescence changes of native and heat-treated (72 °C and 95 °C) BLF and CLF were characterized on a Cary Eclipse fluorescence spectrophotometer (Agilent Technologies Trading Co., Ltd., China). Aliquots (1 mL, 0.2 mg/mL) were transferred into quartz cuvettes. Measurements were conducted at 25 °C: excitation 292 nm, emission scan 300–450 nm, excitation/emission slits 2.5 nm, scan speed 240 nm/min (Jiang et al., 2024).

#### 2.3.5 UV–vis spectroscopy analysis

UV spectral variations of native and heat-treated (72 °C and 95 °C) BLF and CLF were measured using a UV-4802 spectrophotometer (Unico (Shanghai), China). Samples (0.2 mg/mL) were loaded into 1 cm quartz cuvettes, with full-wavelength scanning from 200 to 600 nm at room temperature (Chen, Qu, Gras, and Kentish, 2023).

#### 2.3.6 Circular dichroism analysis

Native and heat-treated BLF and CLF samples (0.2 mg/mL) were loaded into 1 mm pathlength cuvettes. Measurements were conducted on a Jasco J − 1500 spectropolarimeter (Jasco International Co., Ltd., Tokyo, Japan) at ambient temperature under continuous nitrogen flow. Scans ranged from 190 to 260 nm at 100 nm/min, with 0.2 nm resolution, 2.0 nm bandwidth and 20 accumulation cycles (Fan et al., 2019). The resulting far-UV CD spectra were analyzed using the BeStSel (Beta Structure Selection) algorithm to estimate the secondary-structure composition of BLF and CLF under different thermal treatments.

#### 2.3.7 Dynamic light scattering (DLS) measurement

The particle size distribution of native and heat-treated BLF and CLF samples at a protein concentration of 0.2 mg/mL was determined using a Zetasizer Pro Blue dynamic light scattering instrument (Malvern Panalytical, UK). All measurements were carried out at 25 °C. The refractive index of the dispersant was set to 1.334, and the refractive index of camel lactoferrin was fixed at 1.450 (Lin et al., 2022). The *Z*-average hydrodynamic diameter (Dz) and polydispersity index (PDI) were recorded as key output parameters. Each sample was prepared with three independent biological replicates, and all results were expressed as mean ± standard deviation.

#### 2.3.8 Molecular dynamics simulation

The crystal structure of BLF (PDB ID: 1BLF) was obtained from the PDB database as the starting structure for simulation. For CLF, its amino acid sequence (UniProt ID: A0A8B8RBG8) was retrieved from the UniProt database, and the three-dimensional structure of CLF was predicted by AlphaFold 2; the quality and rationality of the predicted CLF structural model were further assessed using the SAVES tool. Both BLF and CLF structures were preprocessed via PyMOL software, where all water molecules were deleted and hydrogen atoms were supplemented.

Molecular dynamics (MD) simulations were carried out with YASARA software (v20.14.24, YASARA Biosciences GmbH, Austria). Prior to simulation, protonation states of the two proteins were determined by hydrogen bond network optimization and pKa prediction at pH 7.0. Energy minimization was then performed via steepest descent and simulated annealing algorithms to eliminate steric conflicts. Each protein system was solvated within a cubic box with an edge length of 96 Å under the AMBER 14 force field. Independent MD simulations were conducted at 298 K (25 °C), 345 K (72 °C) and 368 K (95 °C). Each simulation lasted 100 ns with an integration time step of 1.25 fs. The conformational stability of BLF and CLF was characterized by calculating root-mean-square deviation (RMSD), radius of gyration (R_g_), and solvent-accessible surface area (SASA) (Martinez-Zacarias et al., 2024).

#### 2.3.9 Statistical analysis

All measurements were performed in triplicate, and data were expressed as mean ± standard deviation. Normality test and homogeneity of variance test were conducted prior to variance analysis. Statistical analysis was implemented by GraphPad Prism 9.4.1 software (GraphPad Software, Inc., USA) using one-way analysis of variance (ANOVA). Multiple pairwise comparisons were performed by Tukey's HSD post-hoc test. A *p value ≤ 0.05* was defined as statistically significant.

## 3 Results and discussion

### 3.1 Optimization and validation of camel lactoferrin purification process

Three cation-exchange elution strategies (linear gradient, two-step stepwise elution, multi-step gradient) were compared to establish a CLF purification workflow from camel whey. Elution profiles are displayed in Fig. S2.

Most acidic camel whey proteins carry negative charges under binding buffer and do not bind to the cation-exchange medium; SDS-PAGE confirmed no CLF existed in flow-through fractions. Weakly bound impurities (mainly lactoperoxidase) were eluted at 40mS/cm (40%Buffer B, 0.4 M NaCl) with trace CLF only. The target fraction was harvested at 50mS/cm (100%Buffer B, 1 M NaCl), matching the high theoretical net positive charge of CLF. Linear and multi-step gradients induced severe peak broadening, whereas two-step elution (washing impurities with 40%Buffer B before target elution by 100%Buffer B) produced sharp high-purity CLF peaks. SDS-PAGE of the fractions eluted with 100% Buffer B displayed only a single band. Subsequent LC-MS/MS analysis further validated camel lactotransferrin as the major constituent (Fig. S3 and Table S1). Collectively, these results verified the identity of the target fraction and laid a basis for subsequent optimization of elution parameters.

#### 3.1.1 Preparation of camel lactoferrin reference standard

CLF purified by heparin affinity chromatography exhibited a single band in SDS-PAGE (Fig. S5a). Its identity was validated via LC-MS identification (Fig. S4) with the matched UniProt ID A0A8B8RBG8, and UPLC detection (Fig. S5b) confirmed its high purity as a reference standard. Calibration curves of serial CLF concentrations showed favorable linearity with R^2^ > 0.99 (Fig. S5c and d), supporting accurate quantification of lactoferrin in raw camel whey.

#### 3.1.2 Single-factor study on camel lactoferrin separation by ion exchange chromatography

The effect of mobile phase flow rate on CLF purity was illustrated in Fig. 1a. CLF purity increased significantly (*P < 0.05*) as flow rate rose from 0.50 to 1.00 mL/min and reached the maximum at 1.00 mL/min; further increasing the flow rate to 1.25 and 1.50 mL/min significantly reduced purity (*P < 0.05*). This tendency was supported by SDS-PAGE results (Fig. S6). At 0.50 mL/min, long residence time caused extensive co-adsorption of impurities; 1.00 mL/min yielded nearly pure CLF with a single dominant band. When flow rate exceeded 1.25 mL/min, insufficient interaction between CLF and the stationary phase led to premature target elution and re-emerging impurities. Low flow rates (<0.75 mL/min) ensured sufficient protein adsorption but greatly decreased throughput. Collectively, the optimal flow rate balancing purity and efficiency ranged from 0.75 to 1.25 mL/min. Among these conditions, 1.00 mL/min achieved the highest CLF purity without prolonged separation and was selected for subsequent experiments.

**Fig. 1:**
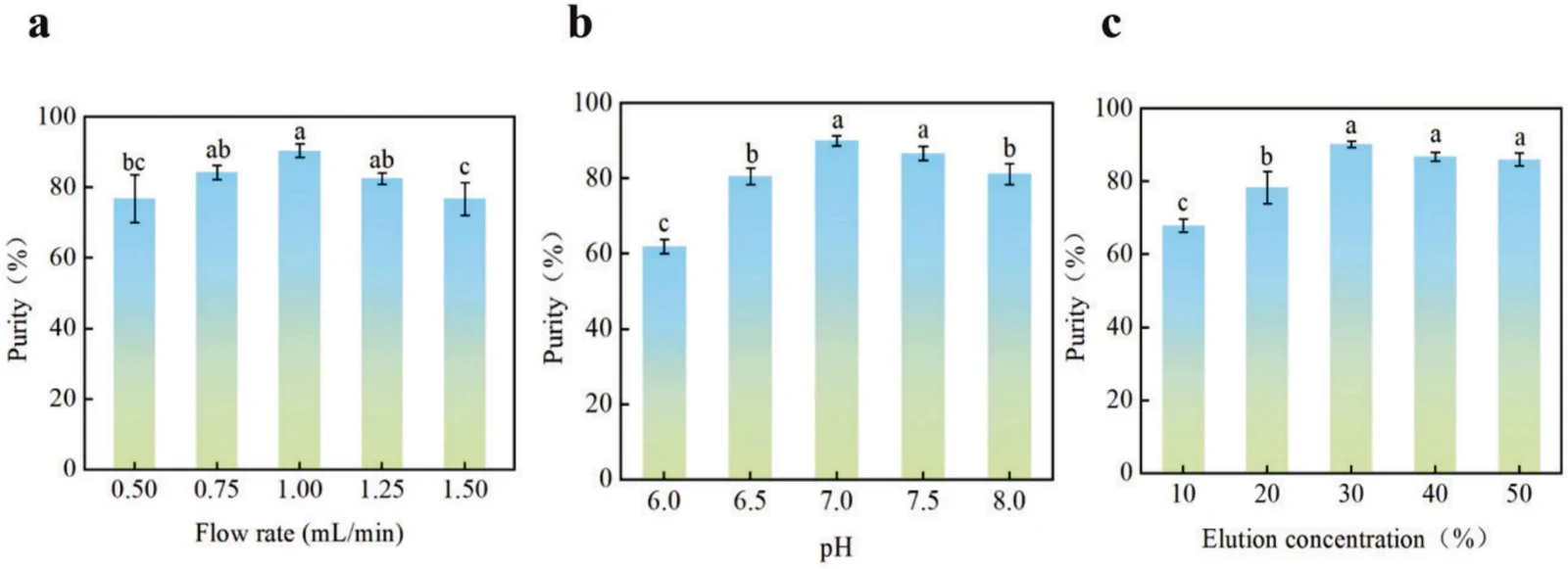
Effect of one-factor buffer flow rate, pH, and elution gradient on CLF purity. (a) Influence of mobile phase flow rate on the final purity of CLF, (b) The effect of buffer pH on the final purity of CLF, and (c) Effect of impurity elution concentrations on the final purity of CLF. (Error bars represent the standard deviation of three replicate experiments; different letters indicate significant differences between groups, *P* < 0.05).

The quantitative results illustrating the effect of buffer pH on CLF purity were shown in Fig. 1b. CLF purity at pH 6.0 and 6.5 was significantly lower than that at pH 7.0 and 7.5 (*P < 0.05*), whereas purity declined at pH 8.0 without significant difference from pH 6.5 (*P > 0.05*). This tendency was verified by SDS-PAGE (Fig. S7): multiple impurity bands emerged at pH 6.0–6.5; samples at pH 7.0–7.5 exhibited nearly pure CLF, and impurities reappeared at pH 8.0. These pH-dependent differences followed the electrostatic adsorption mechanism of ion-exchange chromatography (Petrilli et al., 1977). pH modulated protein net surface charge and binding affinity to the stationary phase. Under acidic conditions, CLF and impurities carried positive charges and co-eluted. Near-neutral pH weakened the binding of acidic impurities, enabling effective separation. At pH 8.0, the pH approached the theoretical pI of CLF, decreased protein net charge and weakened matrix binding, thus reducing purity. Based on purity and electrophoresis data, the optimal buffer pH range of 6.5–7.5 was identified to balance purification efficiency and protein stability.

The quantitative results showing the effect of impurity elution buffer (Buffer B) concentration on CLF purity were presented in Fig. 1c. CLF purity at 10% and 20% Buffer B was significantly lower than that at 30%–50% (*P < 0.05*), while purity remained high with no intergroup differences (*P > 0.05*) above 30%. SDS-PAGE (Fig. S8) supported this trend. At concentrations below 30%, impurities were incompletely washed off and co-eluted with CLF. When Buffer B reached 30%–50%, impurities were fully removed in early fractions, yielding a single dominant CLF band. These separation differences followed the salt displacement elution mechanism (Chakraborty et al., 2022). Ionic strength regulated electrostatic interactions between proteins and cation-exchange resin, and salt ions competitively displaced adsorbed proteins for stepwise separation. Although 30%–50% Buffer B produced high-purity CLF, 50% Buffer B increased the workload of subsequent desalting. Balancing purification performance and desalting burden, the optimal Buffer B concentration range was determined as 30%–40%.

#### 3.1.3 Response surface optimization for CLF purification via ion exchange chromatography

Based on single-factor results, three key parameters were chosen for optimization: buffer flow rate (A), buffer pH (B), and impurity elution concentration (C). Response surface methodology (RSM) and a Box-Behnken design (BBD) were applied to investigate factor interactions and build a regression model correlating chromatographic parameters with CLF purity (Y). A quadratic polynomial equation was obtained via multiple regression:

(2) $$Y=89.84–0.1250A+1.15B+2.68C+0.6750\mathrm{AB}-0.9250\mathrm{AC}-0.8250\mathrm{BCE}-3.71A^{2}–1.76B^{2}–3.76C^{2}$$

The regression model showed satisfactory fitting performance (Table 3). The non-significant lack-of-fit value (*p* = 0.3214, *P > 0.05*) indicated no systematic deviation. With a residual mean square of 0.4035 and standard deviation (SD) of 0.6946, the experiment exhibited low random error. High coefficient of determination (R^2^ = 0.9849) and adjusted R^2^ (R^2^_adj_ = 0.9654) onfirmed reliable fitting, and the model explained 98.49% of total purity variation. Analysis of variance revealed significant effects of B, C, interaction terms AC, BC and all quadratic terms. Factor influence ranked: impurity elution concentration (C) > buffer pH (B) > buffer flow rate (A). Consistent with ion-exchange theory, C regulated ionic strength to control co-elution, pH altered protein surface charge, while flow rate only changed contact time and exerted the weakest influence. Three-dimensional response surfaces and contour plots are displayed in Fig. 2a–c.

**Table 3:** Analysis of variance for CLF purity.

	Sum of squares	Degrees of freedom	Mean square	*F*-value	*p*-value
Model	219.74	9	24.42	50.61	<0.0001
A	0.1250	1	0.1250	0.2591	0.6264
B	10.58	1	10.58	21.93	0.0023
C	57.25	1	57.25	118.66	<0.0001
AB	1.82	1	1.82	3.78	0.0930
AC	3.42	1	3.42	7.09	0.0323
BC	2.72	1	2.72	5.64	0.0492
A^2^	57.88	1	57.88	119.97	<0.0001
B^2^	13.01	1	13.01	26.96	0.0013
C^2^	59.45	1	59.45	123.23	<0.0001
Residual	3.38	7	0.4035		
Lack of Fit	1.85	3	0.6642	1.61	0.3214
Pure Error	1.53	4	0.2080		
Cor Total	223.12	16			
R^2^	0.9849				
R_adj_^2^	0.9654				
SD	0.6946

**Fig. 2:**
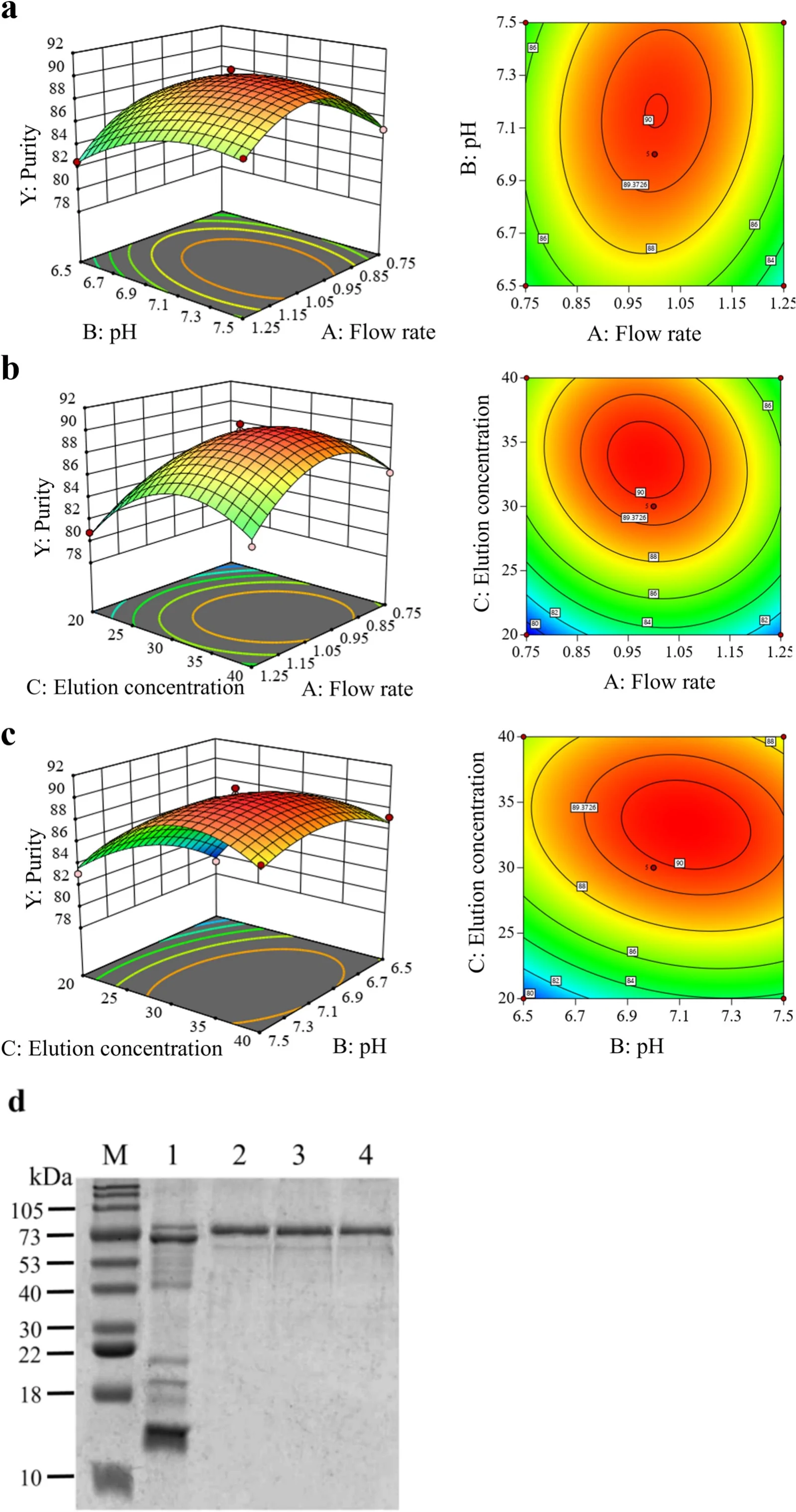
Effect of interaction between factors on CLF purity. (a) 3D response surface (left) and 2D contour plots (right) of the interaction between buffer flow rate (A) and buffer pH (B), (b) 3D response surface (left) and 2D contour plots (right) of the interaction between buffer flow rate (A) and impurity elution concentration (C), and (c) 3D response surface (left) and 2D contour plots (right) of the interaction between buffer pH (B) and impurity elution concentration (C). (d) SDS-PAGE diagram of the optimal condition for purifying CLF. (M represents the Marker. 1 is whey, and 2, 3, and 4 are the results of three purifications under this condition.)

The model predicted optimal conditions: flow rate 1.01 mL/min, pH 7.09, impurity elution concentration 32.78%, with a maximum predicted purity of 90.4%. For operation convenience, parameters were adjusted to 1 mL/min, pH 7.0 and 30% eluent (0.3 MNaCl). The purity of the final CLF preparation reached 90.13 ± 1.06% (Fig. 2d), agreeing closely with the prediction. These results verified that Eq. (2) possessed good predictive capacity for CLF purification optimization.

#### 3.1.4 Validation of lactoferrin purification process

The validation results for the cation-exchange chromatographic purification of CLF are summarized in Table S3. The relative standard deviations (RSD) for precision, repeatability, stability, and spiked recovery were 3.74%, 1.74%, 1.49%, and 2.73%, respectively, all below 5%, indicating excellent reproducibility and robustness of the process. The definition, experimental conditions and calculation formula of spiked recovery are provided in the note under Table S3. Furthermore, the standard curve relating LF peak area to concentration (Fig. S9) showed a linear regression within the range of 0–1 mg/mL: y = 405.93× - 6.8548, with a correlation coefficient (R^2^) of 0.9989 (*>0.99*). This further confirms the reliability of the method for both the purification and detection of LF, meeting the requirements for analytical applications.

### 3.2 Thermal stability analysis and structural characterization of lactoferrin

#### 3.2.1 Iron saturation measurement

The iron-saturation levels of purified BLF and CLF were 27.19% and 33.38%, respectively, with a difference of only 6.19 percentage points. Both proteins are naturally partially iron-saturated lactoferrins falling within the low-to-moderate saturation range. Previous studies have demonstrated that iron exerts the most pronounced effects on lactoferrin conformation and thermal stability during the apo-to-holo transition. Consistently, partially iron-saturated lactoferrin samples with closely comparable saturation degrees generally exhibit similar overall structural and thermal-stability features (Canbolat et al., 2026; Xia et al., 2023). Nevertheless, to what extent this modest 6.19-percentage-point difference in iron saturation could affect thermal-related properties remains unclear for BLF and CLF. Given their comparable native iron-saturation status, we compared the thermal-stability and structural characteristics of BLF and CLF under their naturally partially iron-saturated conditions.

#### 3.2.2 Differential scanning calorimetry (DSC)-based thermal stability analysis

Differential scanning calorimetry (DSC) characterized the thermal denaturation of BLF and CLF (Fig. 3a). Both proteins exhibited two endothermic peaks assigned to sequential unfolding of the N-lobe and C-lobe (Bengoechea et al., 2011). BLF displayed domain melting temperatures (T_m_) of 72 °C and 89 °C, consistent with previous literature (Bokkhim et al., 2013), whereas corresponding T_m_ values of CLF were 67 °C and 91 °C. CLF N-lobe showed a lower T_m_, indicating poorer thermostability; its C-lobe possessed a higher T_m_ and stronger heat resistance. The two proteins thus exhibited opposite thermal trends for their N- and C-lobes, which probably stemmed from species-specific molecular features such as amino acid composition, rigid region distribution and native tertiary folding (Dyrda-Terniuk & Pomastowski, 2023).

**Fig. 3:**
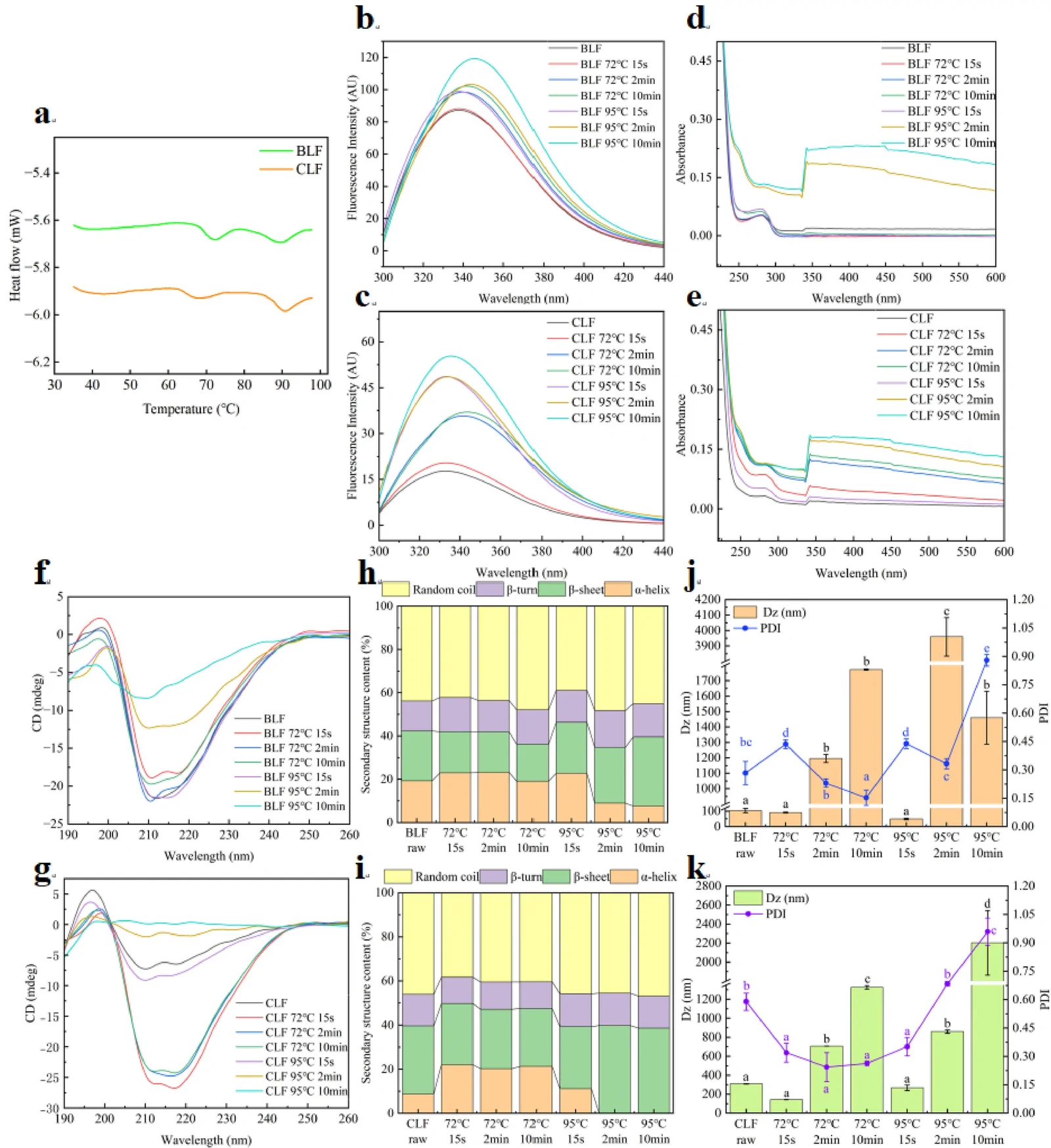
Results of thermal stability and structural characterization of lactoferrin. (a) Differential scanning calorimetry curves of BLF and CLF. (b) Fluorescence spectra of BLF before and after different heat treatments. (c) Fluorescence spectra of CLF before and after different heat treatments. (d) UV–Vis spectra of BLF before and after different heat treatments. (e) UV–Vis spectra of CLF before and after different heat treatments. (f) Circular dichroism (CD) spectra of BLF before and after different heat treatments. (g) Circular dichroism (CD) spectra of CLF before and after different heat treatments. (h) Secondary structure proportions calculated from CD spectra of BLF before and after different heat treatments. (i) Secondary structure proportions calculated from CD spectra of CLF before and after different heat treatments. (j) Changes in average hydrodynamic diameter (Dz) and polydispersity index (PDI) of BLF under various heat treatments. (k) Changes in average hydrodynamic diameter (Dz) and polydispersity index (PDI) of CLF under various heat treatments. Raw groups correspond to native untreated protein samples. Columns represent the average hydrodynamic diameter Dz (nm), and the line plots with circular markers stand for PDI values. Different lowercase letters denote significant differences between treatments (*p* < 0.05).

#### 3.2.3 Fluorescence spectroscopy analysis

Fluorescence spectra of thermally treated BLF and CLF are shown in Fig. 3b and c. Red shifts of maximum emission wavelength were detected for both proteins after heating, indicating changes in the microenvironment around buried aromatic residues (Wang et al., 2022). At 72 °C for 15 s-10 min, both proteins showed slight red shifts accompanied by gradual increases in fluorescence intensity, suggesting relatively mild perturbation of their tertiary structures. At 95 °C, prolonged heating resulted in more pronounced fluorescence changes, indicating substantial perturbation of the local environment surrounding tryptophan residues, with different responses observed between the two proteins. The apparent fluorescence intensity of CLF increased by 212.07%, compared with 36.80% for BLF. Together with the observed changes in emission maximum and overall spectral profiles, these fluorescence responses were consistent with increased exposure of previously buried tryptophan residues and perturbation of their local microenvironment under prolonged high-temperature treatment (Wang et al., 2013). Overall, the spectral changes were more pronounced for CLF, suggesting greater perturbation of the local environment surrounding tryptophan residues. Although both proteins shared similar fluorescence change trends under heating (Konuspayeva et al., 2007), CLF showed more pronounced perturbation of the tryptophan microenvironment after prolonged high-temperature treatment.

#### 3.2.4 UV–vis spectroscopy analysis

UV–vis spectra of thermally treated BLF and CLF were illustrated in Fig. 3d-e. Untreated proteins exhibited characteristic absorption bands within 220–320 nm, with the band near 280 nm mainly arising from aromatic residues such as tryptophan and tyrosine (Cao & Zhao, 2004). After incubation at 72 °C for 15 s, 2 min and 10 min, CLF displayed a greater increase in absorbance around 280 nm than BLF, indicating greater exposure of buried aromatic residues and a stronger hyperchromic effect (Cavallo et al., 2025). Together with the fluorescence results, the UV–vis spectral changes suggest that moderate heating at 72 °C induced relatively mild conformational perturbation in both proteins, with more pronounced changes in the aromatic-residue environment observed for CLF.

Upon heating at 95 °C, both proteins exhibited two concurrent spectral changes with prolonged holding time. Absorbance at 280 nm increased owing to the hyperchromic effect of buried aromatic residues. Continuous baseline elevation within 350–600 nm reflected long-wavelength light scattering, presumably associated with intermolecular aggregation, and both variations became more pronounced with extended thermal treatment. After heating at 95 °C for 2 min and 10 min, CLF showed a smaller extent of baseline elevation in the 350–600 nm region than BLF, indicating weaker long-wavelength light scattering. Taken together with the fluorescence results, these observations suggest that CLF underwent more pronounced perturbation of the aromatic-residue microenvironment and tertiary structure under severe heating, whereas BLF exhibited a stronger long-wavelength scattering response, suggesting the possible formation of larger molecular assemblies.

#### 3.2.5 Circular dichroism (CD) spectroscopy analysis

Circular dichroism (CD) spectroscopy was used to characterize the secondary structural features of BLF and CLF (Fig. 3f–g). Native proteins exhibited similar spectral profiles: a positive peak near 192 nm, a negative band around 210 nm assigned to α-helix, and negative signals in the range of 215–219 nm corresponding to β-sheet, which is consistent with previous reports (Sreedhara et al., 2010). After heating at 72 °C for 10 min, only minor changes were observed in the CD spectra of both proteins, suggesting relatively limited perturbation of their secondary structures. In contrast, heating at 95 °C resulted in pronounced changes in the spectral profiles, including a progressive reduction in negative ellipticity around 210 nm, indicating substantial rearrangement of secondary structure under severe thermal treatment (Fan et al., 2019).

Secondary-structure fitting results presented in Fig. 3h-i revealed both inherent differences between BLF and CLF and treatment-dependent changes in their secondary-structure profiles. Native BLF showed substantial contributions from both α-helix and β-sheet structures, consistent with published data (Xu, 2000), whereas native CLF exhibited a relatively high fitted β-sheet proportion of 30.8%. Under thermal treatment at 72 °C, the fitted secondary-structure fractions of BLF showed only minor variations, indicating relatively limited changes in its secondary-structure organization. By contrast, CLF exhibited more evident redistribution among the fitted secondary-structure components, with the estimated α-helical fraction increasing from 8.8% to over 20%, accompanied by concurrent changes in β-sheet content. Upon heating at 95 °C for 2 min, the fitted α-helical fraction of BLF decreased from 22.8% to 7.6%, whereas its fitted β-sheet contribution increased to 31.9%. CLF likewise showed a marked decrease in the fitted α-helical contribution under the same heating condition, accompanied by a relative increase in β-sheet contribution. The fitted proportion of β-turns remained relatively stable throughout thermal treatment, whereas the estimated random-coil contribution increased moderately with increasing heating intensity, consistent with the spectral changes observed in Fig. 3f–g. Because aggregation-associated light scattering may introduce additional uncertainty into spectral deconvolution under severe heating conditions, these fitted secondary-structure fractions are interpreted primarily in terms of their direction and relative trends of change rather than as absolute quantitative values.

Taken together with the fluorescence and UV–vis results, the CD analysis further indicates that heat-induced structural changes in BLF and CLF involve both tertiary- and secondary-structure perturbations. Notably, CLF exhibited more pronounced structural rearrangement under severe heating, whereas stronger long-wavelength light scattering was observed for BLF in the UV–vis spectra. This contrasting spectroscopic response raises the possibility that BLF and CLF may differ in their tendency to undergo intermolecular association following heat-induced structural perturbation.

#### 3.2.6 Dynamic light scattering (DLS) analysis

Variations in the z-average hydrodynamic diameter (D_z)_ and polydispersity index (PDI) of thermally treated BLF and CLF are shown in Fig. 3j and k. Native BLF and CLF exhibited low D_z_ and PDI values. Heating at 72 °C for 15 s and 2 min had no significant effect on D_z_ (*p > 0.05*); only 10 min heating induced a modest increase in D_z_, indicating limited particle growth. This finding was consistent with the relatively mild conformational and secondary-structure perturbations observed by fluorescence, UV–vis, and CD spectroscopy under moderate heating.

At 95 °C, CLF exhibited moderate increases in D_z_ and PDI with prolonged holding time and maintained lower D_z_ values than BLF across all time points, indicating a lower tendency to form large aggregates. After 10 min, CLF displayed elevated PDI, suggesting an increasingly heterogeneous particle population rather than extensive large-aggregate formation. By comparison, BLF showed a more pronounced particle-size response: treatment for 15 s caused a sharp PDI increase with limited D_z_ growth, indicating a heterogeneous particle population early in thermal treatment (Tomita et al., 2009). At 2 min, BLF reached its maximum D_z_ with decreased PDI, suggesting a larger but relatively more uniform particle population. Further heating to 10 min reduced D_z_ with a slight PDI rebound, indicating redistribution of the particle-size population.

Importantly, the DLS results further clarify the contrasting structural and light-scattering responses observed under severe heating. Although fluorescence and CD analyses indicated more pronounced conformational and secondary-structure perturbation in CLF, its particle-size increase remained relatively limited. In contrast, BLF exhibited substantially greater particle growth, consistent with the stronger long-wavelength light scattering observed in the UV–vis spectra and suggesting a greater tendency of thermally perturbed BLF to form large molecular assemblies. Taken together, these findings further support the possibility raised by the preceding spectroscopic analyses that BLF and CLF differ in their intermolecular association behavior following thermal perturbation.

### 3.3 Molecular dynamics (MD) simulations

To explore thermally induced conformational changes of lactoferrin, MD simulations were performed for BLF and CLF. Residue-level residue root-mean-square fluctuation (RMSF) profiles (Fig. S10) showed that rising temperature markedly increased local flexibility for both proteins, with 95 °C triggering far higher RMSF peaks than 25 °C and 72 °C, indicative of weakened intramolecular interactions and reduced structural stability (Ermakova et al., 2025). Regions with elevated RMSF corresponded to thermally vulnerable segments (Anghel et al., 2018). At 95 °C, CLF displayed a larger RMSF increment than BLF, meaning its structure was more prone to local conformational perturbation. Highly flexible residues mainly concentrated within the N-lobe (residues 0–300) for both proteins, confirming the N-terminal domain was heat-sensitive, whereas the C-lobe exhibited superior thermostability, consistent with our DSC and intrinsic fluorescence results.

Time-dependent root-mean-square deviation (RMSD), radius of gyration (R_g_), solvent-accessible surface area (SASA) and intramolecular hydrogen bond data were summarized in Fig. 4. RMSD reflected backbone deviation from the native structure (Martínez-Zacarias et al., 2024). Under moderate heating (72 °C), both proteins underwent early-stage conformational rearrangement. CLF presented higher RMSD peaks and stronger fluctuations, while BLF experienced milder structural oscillation; nevertheless, their time-averaged RMSD remained comparable. CLF maintained persistently lower R_g_ throughout simulation, revealing a more compact molecular packing. Consistently, after 25 ns, CLF SASA gradually declined alongside stable intramolecular hydrogen bonds in the mid-to-late simulation, whereas BLF exhibited slowly rising SASA and fewer hydrogen bonds. Abundant hydrogen bonding sustained compact geometry, which explained the synchronous decrease of R_g_ and SASA (Schwarcz et al., 2008). At 72 °C, neither protein suffered severe disruption of intramolecular hydrogen bonds.

**Fig. 4:**
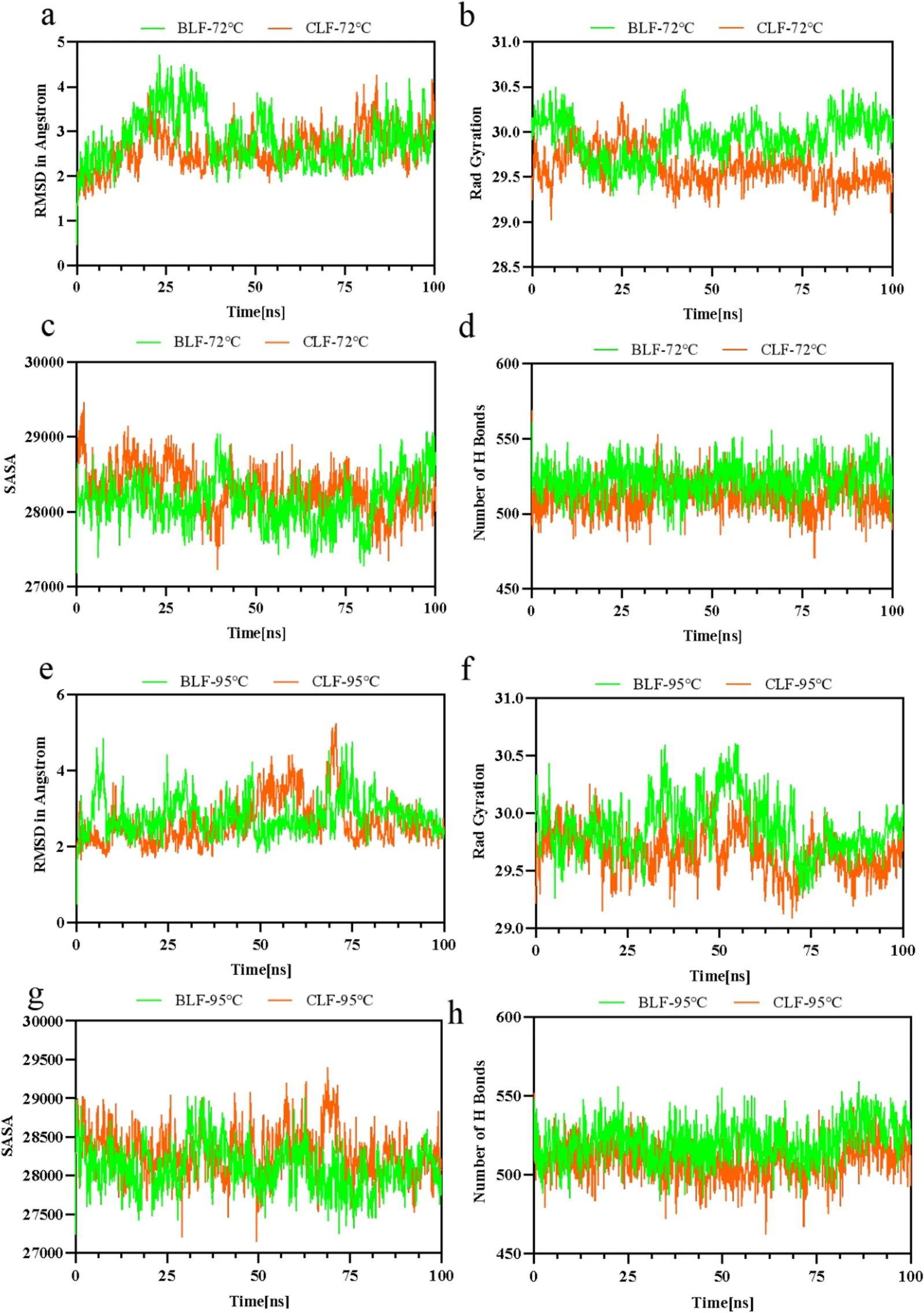
Molecular dynamics simulations of BLF and CLF at 72 °C and 95 °C. (a–d) Changes in RMSD, Rg, SASA, and the number of hydrogen bonds for BLF and CLF at 72 °C. (e–h) Changes in RMSD, Rg, SASA, and the number of hydrogen bonds for BLF and CLF at 95 °C.

Under severe thermal simulation at 95 °C, RMSD values of both proteins rose sharply relative to the 72 °C group, demonstrating that high temperature broke noncovalent interactions and aggravated backbone distortion (Khan, Xiao, et al., 2021). BLF showed higher RMSD maxima and mean values than CLF, representing larger global backbone deviation. Notably, CLF still retained lower R_g_, implying strong internal restraints preserved its compact architecture upon heating. Although CLF formed fewer hydrogen bonds than BLF, we speculated alternative noncovalent interactions (salt bridges, hydrophobic contacts) compensated for hydrogen bond loss to maintain its compact three-dimensional scaffold.

It should be emphasized that compact global morphology (low R_g_) only reflected macroscopic molecular dimension and cannot represent local residue stability. This apparent contradiction could be interpreted by RMSF and fluorescence results: only the CLF C-lobe possessed good thermostability, while its N-lobe was heat-labile. At high temperature, CLF N-terminal residues gained drastically enhanced flexibility, triggering exposure of buried hydrophobic residues and more pronounced local conformational perturbation compared with BLF.

Dynamic evolution of secondary structures at 25 °C, 72 °C and 95 °C was shown in Fig. 5. Heating disrupted intramolecular hydrogen bonds and promoted the transition from ordered secondary structures to random coil. At identical temperatures, CLF always contained a higher fraction of random coil and less α-helix and β-sheet than BLF, accompanied by a more remarkable growth in random coil upon heating. This indicated CLF suffered greater secondary structural disorder during thermal treatment, consistent with RMSF and fluorescence findings. Collectively, despite maintaining a compact global conformation via noncovalent interactions, CLF underwent substantial decay of internal secondary structural order upon heating, featuring a unique thermal pattern: compact overall morphology with disordered interior domains.

**Fig. 5:**
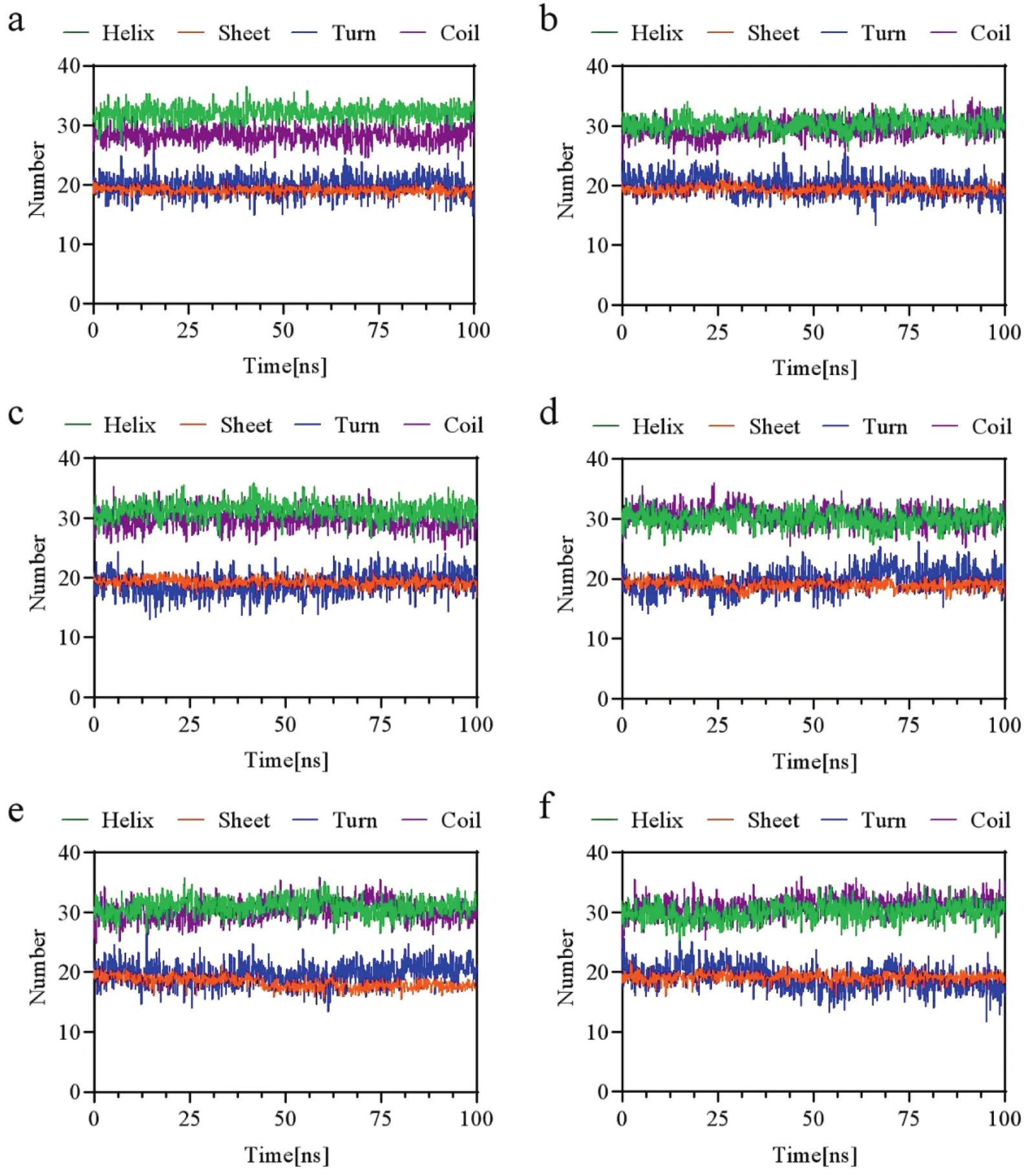
The changes in secondary structure of BLF and CLF at 25 °C, 72 °C, and 95 °C. (a, c, e and b, d, f represent the simulations of BLF and CLF at 25 °C, 72 °C, and 95 °C respectively).

## 4 Conclusion

In this study, cation-exchange chromatography was optimized for the purification of CLF, and the optimal processing parameters were determined as follows: a flow rate of 1 mL/min, buffer pH 7.0, and an impurity elution salt concentration of 30% (0.3 M NaCl). Under these conditions, the purity of the final CLF preparation reached 90.13% ± 1.06%. This purification method exhibits outstanding repeatability and reliability (relative standard deviation, RSD < 5%), and provides a chromatographic approach with potential for industrial processing as well as quantitative analytical use.

Combining multi-scale solution structural characterizations and molecular dynamics simulations, the distinct thermal response behaviors of BLF (native-state iron saturation: 27.19%) and CLF (native-state iron saturation: 33.38%) were systematically deciphered. DSC measurements revealed divergent thermostability of their lobe domains: the N-lobe of CLF exhibited a lower melting temperature (T_m_ = 67 °C) than that of BLF (T_m_ = 72 °C), indicating weaker thermostability, whereas the C-lobe of CLF exhibited a slightly higher melting temperature (91 °C) than that of BLF (89 °C). On this basis, intrinsic fluorescence, UV–vis, and circular dichroism analyses indicated that CLF underwent more pronounced conformational perturbation and secondary-structure rearrangement under severe heating, accompanied by marked changes in ordered secondary-structure components and greater exposure of previously buried aromatic residues. Nevertheless, further analysis of DLS data showed that CLF maintained a lower hydrodynamic diameter (D_z_) and exhibited milder aggregation growth than BLF under identical heating conditions. To interpret this observation, we proposed a plausible mechanism: CLF adopts a characteristic conformation with a compact global architecture yet disordered interior under high temperature. The exposed hydrophobic moieties tend to be shielded via intramolecular interactions, which suppress intermolecular association. By contrast, thermally perturbed BLF exhibited a greater tendency toward intermolecular association and the formation of large molecular assemblies, consistent with its substantially greater particle growth under the same heating conditions.

Molecular dynamics simulations further supported this hypothesis: CLF displayed greater residue flexibility (RMSF) and a more pronounced increase in random coil fraction at high temperatures, while its radius of gyration (R_g_) and SASA remained at relatively low levels. Compensatory intramolecular forces such as salt bridges and hydrophobic interactions stabilize its compact spatial framework. Taken together, these experimental and computational results indicate that protein aggregation propensity is not determined solely by the extent of thermally induced structural perturbation, but may also be influenced by the conformational state and intermolecular association behavior of the thermally perturbed proteins.These divergent thermal responses provide important experimental evidence supporting species-specific thermal denaturation pathways of the two lactoferrins.

In summary, this study established an efficient and stable CLF purification protocol and revealed the molecular mechanism responsible for its unique thermal behavior under laboratory-simulated heating conditions, offering fundamental theoretical support for understanding CLF performance in thermally processed dairy and functional food systems. Further investigations will address the bioactivity retention of thermally treated CLF and its conformational and functional changes in complex milk matrices, which can guide future research toward the exploitation of CLF in high-value dairy products including infant formula and sports nutrition foods.

## CRediT authorship contribution statement

**Yuying Liu:** Writing – review & editing, Writing – original draft, Methodology. **Ying Liu:** Writing – review & editing, Formal analysis, Data curation, Conceptualization. **Hongyi Zhang:** Methodology, Data curation. **Minaer Huanbieke:** Supervision, Methodology, Conceptualization. **Lixiang Ma:** Investigation, Formal analysis, Data curation. **Chunyan Ran:** Supervision, Methodology, Conceptualization. **Yaqi Cheng:** Investigation, Formal analysis, Data curation. **Jie Yang:** Supervision, Funding acquisition. **Haitao Yue:** Supervision, Funding acquisition. **Yang Qu:** Writing – review & editing, Validation, Supervision, Conceptualization.

## Declaration of competing interest

The authors declare the following financial interests/personal relationships which may be considered as potential competing interests: This manuscript has not been published or presented elsewhere in part or in entirety and is not under consideration by another journal. We have read and understood your journal's policies, and we believe that neither the manuscript nor the study violates any of these. The authors declare that they have no known competing financial interests or personal relationships that could have appeared to influence the work reported in this paper.

## Data Availability

Data will be made available on request.

## Appendix A Supplementary data

Supplementary material Purification flowchart, elution profiles, SDS-PAGE, LC-MS/MS identification, UPLC method validation, calibration curve, and RMSF analyses of camel lactoferrin.
