# Supplementary material for: Optimization of purification process and thermal stability of lactoferrin from camel milk
Source: Food Chem X. 2026 Oct 16;40:104616. doi: 10.1016/j.fochx.2026.104616 (PMC13698081; doi:10.1016/j.fochx.2026.104616)
Supplement: Supplementary file 1 [file mmc1.docx]

**Supplementary Material**

Flow diagram of the purification of CLF by cation exchange chromatography

Figure S1. Flow diagram of the purification of CLF by cation exchange chromatography.

Elution Profile and Purity Assessment of CLF Purified by Cation-Exchange Chromatography

Figure S2. Purification of CLF by cation exchange chromatography.

(a).Elution profile of CLF via linear elution following 40% B-phase elution, indicating the flow-through peak and elution peaks.

(b).Elution profile of CLF via sequential elution with 40% B-phase and 100% B-phase, indicating the flow-through peak and elution peaks.

(c).Elution profile of CLF via sequential elution with 40% B-phase, 70% B-phase, and 100% B-phase, indicating the flow-through peak and elution peaks.

(d). SDS-PAGE analysis of CLF purified by cation exchange chromatography (M: protein marker; Lane 1: whey; Lane 2: flow-through peak; Lane 3: eluate from 40% B-phase; Lane 4: eluate from 100% B-phase).

Amino Acid Sequence of Bactripes protease

tr|A0A8B8RBG8|A0A8B8RBG8_CAMFR Lactotransferrin OS=Camelus ferus

OX=419612 GN=LTF PE=3 SV=1

MKLFFPALLSLGALGLCLAASKKSVRWCTTSPAESSKCAQWQRRMKKVRGPS

VTCVKKTSRFECIQAISTEKADAVTLDGGLVYDAGLDPYKLRPIAAEVYGTENNPQTHYYAVAIAKKGTNFQLNQLQGLKSCHTGLGRSAGWNIPMGLLRPFLDWTGPPEPLQKAVAKFFSASCVPCVDGKEYPNLCQLCAGTGENKCACSSQEPYFGYSGAFKCLQDGAGDVAFVKDSTVFESLPAKADRDQYELLCPNNTRKPVDAFQECHLARVPSHAVVARSVNGKEDLIWKLLVKAQEKFGRGKPSAFQLFGSPAGQKDLLFKDSALGLLRIPSKIDSGLYLGSNYITAIRGLRETAAEVELRRAQVVWCAVGSDEQLKCQEWSRQSNQSVVCATASTTEDCIALVLKGEADALSLDGGYIYIAGKCGLVPVLAESQQSPESSGLDCVHRPVKGYLAVAVVRKANDKITWNSLRDKKSCHTAVDRTAGWNIPMGLLFKNTDSCRFDEFFSQSCAPGSDPRSKLCALCAGNEEGQNKCVPNSSERYYGYTGAFRCLAENVGDVAFVKDVTVLDNTDGKNTEQWAKDLKLGDFELLCLNGTRKPVTEAESCHLAVAPNHAVVSRIDKVAHLEQVLLRQQAHFGRNGQDCPGKFCLFQSKTKNLLFNDNTECLAKLQGKTTYEEYLGPQYVT AIAKLRRCSTSPLLEACAFLMR

LC-MS/MS Identification of Cation-Exchange Purified Camel Lactoferrin

Figure S3. Base peak chromatogram (BPC) of peptides from cation-exchange purified camel lactoferrin analyzed by LC-MS/MS

Table S1. Proteins identified from peptides of cation-exchange purified camel lactoferrin analyzed by LC-MS/MS

| Majority protein IDs | Protein names | Gene names | Number of proteins | Peptides | Razor + unique peptides | Unique peptides | Sequence coverage [%] | Mol. weight [kDa] | Sequence length | Score |
| --- | --- | --- | --- | --- | --- | --- | --- | --- | --- | --- |
| A0A9W3F6J9 | Lactotransferrin | LTF | 1022 | 144 | 144 | 10 | 47.5 | 77.36 | 708 | 323.31 |
| A0A9W3FK65;A0A077SL35;A0A077B3P4 | Beta-casein;Beta-casein (Fragment) | CSN2; | 93 | 60 | 60 | 59 | 57.6 | 26.028 | 231 | 323.31 |
| A0A9W3F240 | Alpha-lactalbumin | LALBA | 67 | 19 | 19 | 17 | 53.5 | 16.459 | 142 | 323.31 |
| A0A9W3F2R1 | Glycosylation-dependent cell adhesion molecule 1 | LOC105076320 | 171 | 58 | 58 | 7 | 61.5 | 22.274 | 200 | 140.61 |
| A0A9W3F965 | Alpha-S1-casein | CSN1S1 | 208 | 28 | 28 | 26 | 47.7 | 25.843 | 222 | 134.22 |
| A0A9W3EWK0 | Amine oxidase | LOC105072853 | 51 | 41 | 41 | 38 | 39.9 | 58.62 | 521 | 91.328 |
| A0A9W3GDF4;A0A9W3EXF2;A0A9W3GDD5;A0A9W3HKV2;A0A9W3HMZ9 | Lipopolysaccharide-binding protein; | LOC105073481; | 15 | 5 | 5 | 4 | 10.3 | 52.877 | 477 | 47.766 |
| A0A9W3EQ51 | Lactadherin | MFGE8 | 1 | 8 | 8 | 8 | 12.6 | 47.656 | 430 | 47.256 |
| A0A9W3EPW3 | Antithrombin-III | SERPINC1 | 1 | 8 | 8 | 8 | 14.6 | 52.629 | 465 | 31.298 |
| A0A9W3EK51;A0A9W3FZT7 | Lactoperoxidase | LPO | 156 | 32 | 32 | 28 | 25.6 | 83.691 | 738 | 29.799 |
| A0A9W3F984;A0A9W3FKB4 | Alpha-S2-casein | LOC105080410 | 2 | 9 | 9 | 9 | 28.5 | 22.977 | 193 | 24.242 |
|  |  |  |  |  |  |  |  |  |  |  |
|  |  |  |  |  |  |  |  |  |  |  |
| W0K478;L0P304 | Kappa-casein (Fragment);Kappa-casein | ;csn3 | 14 | 28 | 28 | 24 | 51.6 | 17.155 | 153 | 22.877 |
| A0A9W3H8F1;A0A9W3F8P5;A0A9W3FK77;A0A9W3H825;A0A9W3FK33;A0A9W3FKJ3;A0A9W3FKM9;A0A9W3FK69;A0A9W3FK81;A0A9W3H782 | Inter-alpha-trypsin inhibitor heavy chain H4 isoform X7;Inter-alpha-trypsin inhibitor heavy chain H4 isoform X6 | ITIH4; | 10 | 6 | 6 | 6 | 7.6 | 105.7 | 955 | 20.448 |
| A0A9W3H6E1;A0A9W3H6J4 | Lactotransferrin | LTF | 2 | 137 | 3 | 3 | 49.2 | 75.04 | 687 | 18.814 |
| A0A9W3GQ90 | Neutrophil gelatinase-associated lipocalin | LCN2 | 1 | 3 | 3 | 3 | 12.8 | 37.594 | 335 | 17.094 |
| A0A9W3F5K3 | Complement C3 | C3 | 1 | 7 | 7 | 7 | 4.9 | 187.31 | 1664 | 15.156 |
| A0A9W3FEA5 | Fibroblast growth factor-binding protein 1 | FGFBP1 | 12 | 8 | 8 | 6 | 13.5 | 26.836 | 237 | 10.249 |
| A0A9W3FTI0 | Platelet glycoprotein 4 | LOC105062383 | 2 | 4 | 4 | 4 | 7.6 | 52.681 | 472 | 9.3963 |
| A0A9W3HDU9 | Uncharacterized protein LOC105065413 | LOC105065413 | 1 | 2 | 2 | 2 | 4.9 | 42.032 | 384 | 8.4291 |
|  |  |  |  |  |  |  |  |  |  |  |
|  |  |  |  |  |  |  |  |  |  |  |
| A0A9W3GQV8 | Fatty acid-binding protein, heart | FABP3 | 1 | 3 | 3 | 3 | 19.5 | 14.692 | 133 | 7.8149 |
| A0A9W3EPQ9 | Sulfhydryl oxidase | QSOX1 | 1 | 8 | 8 | 7 | 8.4 | 78.684 | 717 | 7.6038 |
|  |  |  |  |  |  |  |  |  |  |  |
| A0A9W3H822;A0A9W3FJE6;A0A9W3FJJ3;A0A9W3H7Q1;A0A9W3H281;A0A9W3FCP4;A0A9W3H1J2 | Serpin A3-8 isoform X5;Serpin A3-8 isoform X4; | LOC105079903; | 7 | 4 | 4 | 3 | 10 | 44.78 | 401 | 6.2449 |
| A0A9W3HAW8;A0A9W3GLZ6 | Alpha-2-macroglobulin-like isoform X1;Alpha-2-macroglobulin-like isoform X2 | LOC105062185 | 2 | 7 | 7 | 6 | 5.3 | 148.74 | 1343 | 6.0586 |
| A0A9W3F4V6 | Osteopontin | SPP1 | 12 | 5 | 5 | 4 | 11.8 | 33.907 | 305 | 6.0554 |
| A0A9W3GMC1 | Peptidoglycan-recognition protein | PGLYRP1 | 1 | 6 | 6 | 5 | 20.7 | 21.349 | 193 | 5.429 |
| A0A9W3F988 | Albumin | ALB | 216 | 17 | 16 | 13 | 16.6 | 69.399 | 607 | 5.3958 |
| A0A9W3ERW7 | Pepsin A | LOC105070098 | 1 | 7 | 7 | 7 | 13.8 | 42.131 | 390 | 2.9344 |
| A0A9W3GIG8 | Glycosylation-dependent cell adhesion molecule 1 | LOC105076320 | 1 | 52 | 2 | 2 | 58.4 | 20.493 | 185 | 2.3493 |
|  |  |  |  |  |  |  |  |  |  |  |
|  |  |  |  |  |  |  |  |  |  |  |
| A0A9W3EXK5 | Haptoglobin | LOC105073420 | 1 | 3 | 3 | 3 | 6.1 | 38.501 | 347 | 1.5835 |
| A0A9W3FG03 | Butyrophilin subfamily 1 member A1 isoform X3 | LOC105084142 | 3 | 3 | 3 | 3 | 7 | 59.038 | 526 | 1.5225 |
| A0A9W3EG71 | Clusterin | CLU | 1 | 3 | 3 | 3 | 8.5 | 51.428 | 445 | 1.4686 |
| A0A9W3GSV6 | Peptidyl-prolyl cis-trans isomerase | PPIB | 2 | 3 | 3 | 3 | 11.1 | 23.857 | 216 | 0.80756 |
| A0A9W3GZ61 | Mucin-15 | MUC15 | 1 | 4 | 4 | 4 | 15.1 | 35.641 | 332 | 0.46577 |
| A0A9W3FT22 | Pregnancy zone protein | LOC105062187 | 1 | 2 | 2 | 2 | 1.7 | 164.41 | 1478 | 0.37253 |
| A0A9W3H157;A0A9W3FI75;A0A9W3H6T5;A0A9W3FI65 | chymotrypsin | LOC105078637; | 4 | 2 | 2 | 2 | 3.8 | 27.76 | 263 | 0.33669 |
| A0A9W3FKF0;A0A9W3F8W2 | Inter-alpha-trypsin inhibitor heavy chain H1 | ITIH1 | 2 | 2 | 2 | 2 | 3.2 | 91.932 | 824 | 0.22003 |

Note: The number of unique peptides cannot reflect the relative mass abundance of proteins. Camel lactotransferrin (CLF) was confirmed as the predominant component by HPLC purity (90.13%). Other identified proteins are trace residual milk proteins co-eluted during cation-exchange chromatography. Only proteins with ≥2 unique peptides are listed in this table.

LC-MS/MS Identification of Purified Camel Lactoferrin Standard

Figure S4. Total ion current (TIC) profile of purified camel lactoferrin analyzed by LC-MS.

Standard Curve Establishment of Camel Lactoferrin

An appropriate amount of CLF standard purified by heparin affinity chromatography was serially diluted to 100 μg/mL, 125 μg/mL, 250 μg/mL, 500 μg/mL, 750 μg/mL and 1000 μg/mL using the BCA protein quantification kit.

For UPLC quantitative detection of CLF standard solutions, an ACQUITY UPLC system equipped with an ACQUITY UPLC Protein BEH C4 column (300 Å, 1.7 μm, 2.1 mm × 100 mm) was adopted. Mobile phase A was ultrapure water containing 0.1% trifluoroacetic acid, and mobile phase B was acetonitrile containing 0.1% trifluoroacetic acid. The injection volume was 10 μL, flow rate was set at 0.3 mL/min, detection wavelength was 214 nm, and the total separation runtime was 10.24 min. The detailed mobile phase elution gradient is shown in Table S1.

Subsequently, detect the CLF standard solutions at different concentrations with an AKTA Purifier instrument under the same chromatographic conditions as those optimized in the single-factor response surface study. Finally, plot the standard curve of CLF peak area, with CLF standard concentration on the x-axis and the integrated peak area at 280 nm on the y-axis (Figure S7d).

Table S2 Mobile phase elution gradient of ACQUITY UPLC

| Time (min) | Mobile Phase A (%) | Mobile Phase B (%) |
| --- | --- | --- |
| 0 | 70 | 30 |
| 2.08 | 45 | 55 |
| 4.17 | 40 | 60 |
| 5.00 | 70 | 30 |
| 6.67 | 70 | 30 |

Figure S5. Purification of the CLF standard using a heparin affinity column. (a) SDS-PAGE analysis of CLF: lane 1, camel whey; lane 2, CWP fractions 5-7; lane 3, flow-through fraction; lane 4, eluted fraction. (b) Liquid chromatography profile of the purified CLF standard. (c) Liquid chromatography profiles of CLF standards at different concentrations. (d) Standard curve of CLF peak area.

SDS-PAGE Analysis of CLF under Different Mobile Phase Flow Rates

Figure S6. SDS-PAGE analysis of CLF purification under different mobile phase flow rates (the flow rates corresponding to a, b, c, d, and e are 0.5, 0.75, 1.0, 1.25, and 1.5 mL/min, respectively; M represents the protein marker; Lanes 1–4 correspond to camel whey, flow-through fraction, elution peak 1 fraction, and elution peak 2 fraction, respectively).

SDS-PAGE Analysis of CLF under Different Buffer pH Conditions

Figure S7. SDS-PAGE analysis of the CLF purification process at different buffer pH values (a, b, c, d, and e correspond to buffer pH 6.0, 6.5, 7.0, 7.5, and 8.0, respectively; M: Protein molecular weight marker; Lanes 1-4: Camel whey, flow-through fraction, elution peak 1 fraction, and elution peak 2 fraction, respectively).

SDS-PAGE Profiles of CLF as a Function of Impurity Protein Elution Concentration

Figure S8. SDS-PAGE analysis of the CLF purification process at different impurity elution concentrations (a, b, c, d, and e correspond to impurity elution concentrations of 10%, 20%, 30%, 40%, and 50%, respectively; M: Marker; Lanes 1–4: camel whey, flow-through fraction, elution peak 1 fraction, and elution peak 2 fraction, respectively).

Validation of the Lactoferrin Purification Process

Table S3. Measurement of Precision, Reproducibility, Stability, and Spiked Recovery

| No. | Precision | Repeatability | Stability | Spiked Recovery (%) |
| --- | --- | --- | --- | --- |
| 1 | 210.22 | 190.61 | 191.36 | 100.45 |
| 2 | 195.3 | 188.07 | 191.78 | 98.32 |
| 3 | 191.34 | 191.1 | 195.15 | 96.36 |
| 4 | 194.29 | 197.3 | 189.16 | 94.13 |
| 5 | 192.69 | 194.88 | 196.26 | 95.33 |
| 6 | 190.48 | 190.79 | 195.76 | 100.45 |
| RSD（%） | 3.74 | 1.74 | 1.49 | 2.73 |

Note: Spiked recovery in this study refers to matrix standard-addition recovery. A known mass of CLF standard was added into blank 0.02 M PBS buffer (pH 7.0), and the recovered CLF content was quantified based on peak area measured under optimal chromatographic conditions. Calculation formula: Spiked recovery (%) = $\frac{\text{measured mass of recovered CLF }}{\text{theoretical spiked mass of CLF}}$ × 100%

Figure S9.Calibration curve of CLF purified by cation-exchange chromatography

Effect of Temperature on Root Mean Square Fluctuation (RMSF)

Figure S10. Root mean square fluctuations (RMSF) of BLF and CLF at 25℃, 72℃, and 95℃. (a) RMSF of BLF at different temperatures; (b) RMSF of CLF at different temperatures.
